# Effectiveness of an amlodipine and perindopril arginine-based strategy for hypertension management in Morocco: a prospective observational study

**DOI:** 10.11604/pamj.2025.52.186.49213

**Published:** 2025-12-24

**Authors:** Selma Siham El Khayat, El Ghali Mohamed Benouna, Camille Maadjhou Mba, Armand Mbanya, Jean Claude Mbanya

**Affiliations:** 1 Nephrology Department, Ibn Rochd University Hospital, Casablanca, Morocco,; 2 Cardiology Department, Ibn Rochd University Hospital, Casablanca, Morocco,; 3 Health of Population in Transition Research Group, University of Yaoundé I, Yaoundé, Cameroon,; 4 Department of Public Health, Faculty of Medicine and Biomedical Sciences, University of Yaoundé I, Yaoundé, Cameroon,; 5 Department of Internal Medicine and Specialties, Faculty of Medicine and Biomedical Sciences, University of Yaoundé I, Yaoundé, Cameroon

**Keywords:** Adverse effects, amlodipine, blood pressure control, hypertension management, perindopril arginine

## Abstract

**Introduction:**

hypertension is a leading cause of cardiovascular disease and mortality in Morocco, with many patients remaining uncontrolled on monotherapy. This study evaluated the effectiveness and safety of adding perindopril arginine to amlodipine for better blood pressure (BP) control among patients with hypertension in Morocco.

**Methods:**

this multicentre, prospective observational study enrolled 1,614 patients with hypertension who were previously uncontrolled on amlodipine monotherapy and were prescribed perindopril. Baseline characteristics, BP measurements, and adverse events were monitored over 90 days.

**Results:**

participants had a mean age of 63.1 ± 11.2 years, with 66.5% being women. Significant BP reductions were observed from baseline to day 90: mean systolic BP decreased from 171.3 mmHg to 128.7 mmHg (mean difference: -42.6 mmHg, p<0.0001) and mean diastolic BP from 92.4 mmHg to 74.6 mmHg (mean difference: -17.8 mmHg, p<0.0001). BP control (<140/90 mmHg) increased from 60.0% of patients at day 30 to 91.3% at day 90 (p<0.0001). Safety assessments indicated minimal adverse events, with incidence decreasing over the study period. By day 90, only 0.25% of participants reported adverse events, including hypotension (0.06%), fatigue (0.06%), and headaches (0.06%). No serious adverse events were reported.

**Conclusion:**

Moroccan patients with hypertension treated with a combination of amlodipine and perindopril arginine showed a significant improvement in BP control and a favourable safety profile. This strategy presents a promising approach for managing hypertension in similar populations.

## Introduction

The burden of non-communicable diseases (NCDs) is escalating rapidly in Africa, with cardiovascular diseases (CVDs) being the leading cause of death in North African countries, including Morocco. As of 2018, ischemic heart disease accounted for 57% of deaths in Morocco, with hypertension being a significant risk factor, affecting 29.3% of the population; yet, an estimated 75% of those with hypertension do not receive any pharmacological treatment [1,2]. Achieving and maintaining cardiovascular health at the individual level can significantly contribute to reducing the overall burden of CVD in the population [3].

Early and effective blood pressure (BP) control is crucial in preventing cardiovascular and systemic complications. The World Health Organization (WHO) recommends initiating antihypertensive treatment within four weeks of diagnosis if systolic blood pressure (SBP) is ≥140 mmHg or diastolic blood pressure (DBP) is ≥90 mmHg [4]. Long-acting dihydropyridine calcium channel blockers (CCBs) are particularly recommended for patients of African descent [4]. When additional medication is required, an angiotensin-converting enzyme inhibitor (ACEi), angiotensin-receptor blocker, or diuretic is recommended, with single-pill combination therapy preferred for improved adherence and BP control [4].

Morocco aligns with international recommendations, with amlodipine widely utilised as a first-line treatment for hypertension, either alone or in combination with other agents. The single-pill combination of amlodipine and perindopril arginine has market authorisation and is “indicated as substitution therapy for the treatment of essential hypertension and/or stable coronary artery disease, in patients already controlled with perindopril and amlodipine given concurrently at the same dose level” [5]. Amlodipine (a CCB) and perindopril (an ACEi) are effective in reducing BP as a single-pill combination (SPC) more than their respective monotherapies. Meta-analyses and systematic reviews of clinical trials have demonstrated the benefits of this combination in lowering BP, improving medication adherence, and reducing adverse events [6,7]. However, there is limited evidence from real-world observational studies in Northern African populations [8].

This is particularly important given the unique characteristics of hypertension in African populations, including different pharmacogenetic profiles and distinct healthcare delivery challenges [9,10]. Furthermore, most existing studies have focused on single-pill combinations, with limited data on the effectiveness of transitioning from monotherapy to free combinations in real-world clinical practice [11-13]. The Moroccan healthcare context presents specific socio-cultural challenges that may influence treatment outcomes [14]. Understanding how internationally recommended treatment strategies perform in this specific setting is essential for optimising local hypertension management protocols and informing regional health policy decisions.

The study objective was to evaluate the effectiveness and safety of an amlodipine and perindopril arginine-based strategy in patients with hypertension who were uncontrolled on monotherapy of amlodipine and were prescribed perindopril in free combination in a real-world setting in Morocco. We hypothesized that this strategy would be associated with a significant reduction in systolic and diastolic BP after 90 days compared to baseline and would have a favourable safety profile. Specifically, we sought to determine whether the addition of perindopril arginine improves BP control rates across hypertension stages.

To guide the analysis, the study addressed two research questions: 1) Among patients uncontrolled on amlodipine monotherapy, does the addition of perindopril arginine reduce systolic and diastolic BP over 90 days in routine Moroccan clinical practice? 2) What are the BP control rates and safety outcomes associated with this treatment strategy across hypertension stages?

## Methods

**Study design:** this is a multicentre, prospective observational study to evaluate the effectiveness of an amlodipine and perindopril arginine-based strategy in reducing systolic and diastolic blood pressure over 90 days in patients with hypertension who were previously uncontrolled on monotherapy of amlodipine.

**Study setting and population:** the study enrolled patients with hypertension from 89 centres across Morocco between October 2023 and March 2024. The study was conducted in primary care, outpatient settings. Participants were followed for 90 days. Eligible patients were uncontrolled on monotherapy of amlodipine and had perindopril arginine added to their treatment regimen by their primary care physicians. Patients were seen monthly (day 30, day 60, and day 90) after initiation of the amlodipine and perindopril arginine-based strategy by the treating physician, per WHO recommendations [4]. Once controlled, patients receiving a free combination treatment of amlodipine and perindopril arginine could be switched to a single-pill combination based on their physician´s judgement. All therapeutic decisions were made based on the physician´s judgement in accordance with the national protocol for hypertension management. Participants could see their treating physician as needed.

**Sampling strategy:** physicians at each participating centre screened adults receiving routine hypertension consultations for study eligibility based on inclusion and exclusion criteria. Eligible patients whose physicians independently decided to add perindopril arginine to their amlodipine hypertension regimen were invited to participate. Consecutive sampling was done.

**Inclusion criteria:** study participants were men or women aged ≥18 years who provided written informed consent. Eligible participants were patients with hypertension previously treated with amlodipine in a monotherapy regimen who remained uncontrolled and for whom the treating physician decided to add perindopril arginine in free combination.

**Exclusion criteria:** patients were excluded if they were pregnant, breastfeeding, or had the possibility of becoming pregnant during the study. Patients in another randomised study within the preceding 3 months were excluded. Other exclusion criteria included known symptomatic orthostatic hypotension, hyperkalaemia or hypokalaemia, history of hypertension resistant to free or single-pill combinations of perindopril and calcium channel inhibitors, contraindications to treatment with perindopril or amlodipine, secondary or complicated hypertension, known renal impairment, chronic pancreatitis, complicated liver disease, recent ventricular rhythm disorders, and a history of heart or cerebrovascular diseases.

**Variables:** the study's primary outcome was the mean change in office systolic blood pressure (SBP) and diastolic blood pressure (DBP) between baseline and the end of the study (day 90). Secondary outcomes included BP control defined as <140/90 mmHg, treatment adherence as self-reported by patients to the treating physician, and the occurrence of adverse events during follow-up reported by the patient or identified by the physician. Patient characteristics captured were age, sex, and smoking status at enrolment, as well as medical history of significant conditions, diseases, and medications relevant to the study. Clinical variables included baseline blood pressure, heart rate, and concomitant medications, as well as ambulatory blood pressure if available. Safety variables were adverse events reported or identified through the physician´s assessment at each visit. Confounding variables for adjusted models included age, sex, duration of hypertension, smoking status, reported physical activity, diet, BMI, and comorbidities (type 1 and type 2 diabetes, kidney disease, cardiovascular disease, and dyslipidaemia). Confounders were selected a priori based on clinical relevance and established associations with BP outcomes in the hypertension literature.

**Data sources and measurement:** demographic and clinical information, including date of birth or age, sex, and smoking status at enrolment, and relevant medical history and concomitant medications were documented during a physician visit. Medical history obtained determined if the subject had any significant conditions or diseases relevant to the study that ceased at or before signing the informed consent. Information on concomitant medication included any relevant medications stopped within 30 days prior to signing the informed consent.

Vital signs collected at each physician visit included respiratory rate, SBP, DBP, and heart rate (bpm). Blood pressure values were defined by office measurements using an automatic device, after at least a 10-minute rest in a sitting position. Blood pressure measurements were done according to the European Society of Hypertension guidelines [15]. Ambulatory blood pressure monitoring was documented if available. Patients spontaneously reported adverse events, and/or the treating physicians directly questioned and examined their patients for adverse events at each study visit. Adherence to antihypertensive treatment was assessed through self-reported responses to questions from the treating physician on missed doses, and intentional non-adherence, including stopping when feeling better, or due to adverse effects. A standardized measurement protocol was recommended to physicians across all study sites. Device models varied by site due to real-world practice conditions. Data was collected and entered by treating physicians during clinical visits.

**Study size:** the sample size was calculated using the standard formula for detecting a mean difference in a continuous outcome [16]:

where n=required sample size; Z_1-α/2_=Z-score for two-sided significance level (α=0.05), Z_1-β_=Z-score for power (95%); σ= standard deviation of the outcome; δ= expected mean difference to detect. We specified a clinically relevant reduction in SBP, and its standard deviation based on prior data and inflated the resulting estimate by 20% to account for potential loss to follow-up. This yielded a minimum required sample size of 1,440 participants, and we therefore aimed to enrol 1,600 patients.

**Statistical methods:** to assess effectiveness, we compared changes in office SBP and DBP across study visits using paired tests and estimated adjusted effects using linear mixed-effects models. To assess safety, we summarized adverse events at each visit and compared their frequency over time.

Results are presented as arithmetic mean ± standard deviation or median (interquartile range) for normally or non-normally distributed continuous variables, respectively. Categorical variables are presented as numbers and percentages. For comparing variables between two study visits, a McNemar´s test was used for categorical variables, a Wilcoxon signed rank test for non-normally distributed continuous variables, and a paired t-test for normally distributed continuous variables. The effects of covariates were estimated using regression coefficients obtained from linear mixed effects models. No sensitivity analyses were performed. All participants who did not complete the study were included in the analyses. Random imputations were done for missing data points, under the assumption that data were missing at random. All statistical analyses were performed using Stata (15, StataCorp LLC, College Station, TX). The null hypothesis was always rejected for p<0.05.

**Ethical considerations:** the study protocol was approved by the Comité d´Ethique pour la Recherche Biomédicale de Rabat (Protocol no. IC4-05985-014-MAR), and procedures were performed in accordance with the Helsinki Declaration of 1964, and its later amendments. All patients provided written informed consent to participate and could discontinue the study at any time and for any reason.

## Results

**Figure 1:**
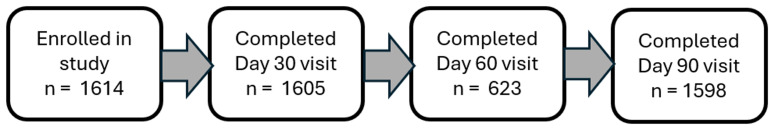
flow chart showing study participation across study visits

**Participants:** a total of 1,614 patients with hypertension were enrolled in the study. The overall withdrawal rate was 0.99% by day 90. Reasons for study withdrawal included loss to follow-up and death (investigated as per the approved study protocol and reported without being related to the medication) (Figure 1).

**Descriptive data:** the mean age of the participants was 63.1 ± 11.2 years. Most participants were women (66.5%). The median duration of known hypertension was 6 years. Regarding smoking status, 82.4% had never smoked, 9.5% were past smokers, and 8.1% were current smokers. The mean duration of smoking for current smokers was 24.1 ± 12.6 years, and the median duration of smoking cessation for past smokers was 8 years (5-10 years). Regular physical activity was self-reported by 40.5% of participants. Comorbidities included type 2 diabetes (35.7%, mean duration of 8 years (5-12)), type 1 diabetes (2.7%, mean duration of 11 years (7-16 years)), kidney disease (3.1%), coronary heart disease (0.2%), peripheral artery disease (1.1%), and dyslipidaemia (26.2%) (Table 1).

**Table 1:** baseline characteristics of patients with hypertension uncontrolled on amlodipine monotherapy, enrolled from 89 centres across Morocco between October 2023 to March 2024 (N=1,614)

Characteristics	Results
**Clinical and biological characteristics**	
Weight (Kg)	76.4 ± 13.7
BMI (Kg/m^2^)	27.8 ± 4.9
BMI <18.5	10(0.6)
BMI 18.5-24.9	492(30.5)
BMI 25-29.9	634(39.3)
BMI ≥30	478(29.6)
Office sitting systolic blood pressure (mmHg)	171.3 ± 14.3
Office sitting diastolic blood pressure (mmHg)	92.4 ± 9.6
Office pulse rate (bpm), median [IQR]	79[72-86]
Stage 1	212(13.1)
Stage 2	871(54.0)
Stage 3	531 (32.9)
Home BP measurement, n (%)	527(32.6)
Ambulatory blood pressure measurement, n (%)	39(2.4)
**Blood pressure used to assess control, n (%)**	
Office BP	1431(88.7)
Home BP	171(10.6)
ABPM	12(0.7)
Amlodipine duration (years), median [IQR]	5[2-7]
**Sociodemographic, lifestyle and medical history**	
Age	63.1±11.2
**Sex, n (%)**	
Women	1074(66.5)
Known hypertension (years)	6[3-10]
**Smoking, n (%)**	
Never smoked	1330(82.4)
Past smoker	153(9.5)
Current smoker	131(8.1)
**Smoking type, n (%)**	
Cigarette	254(97.7)
Cigar	2(0.8)
Hookah	4(1.5)
Smoking years for current smokers	24.1± 12.6
Smoking cessation (years)	8[5-10]
Regular physical activity, n (%)	654(40.5)
Known Type 2 diabetes, n (%)	576(35.7)
Known Type 2 diabetes years	8[5-12]
Known Type 1 diabetes, n (%)	43(2.7)
Known Type 1 diabetes years	11[7-16]
Known Kidney disease, n (%)	50(3.1)
Known kidney years	3[2-5]
Known coronary heart disease, n (%)	3(0.2)
Known coronary heart disease years	2.5[2-3]
Known peripheral artery disease, n (%)	17(1.1)
Known peripheral artery disease for years	4[2-9]
Known dyslipidaemia	423(26.2)

Results are presented as arithmetic mean (or median [25^th^-75^th^ percentile] for non-normally distributed variables) or n (%) for categorical variables; *stage 1: BP between 140/90 mmHg and 159/99 mmHg; stage 2: BP between 160/100 mmHg and 180/120 mmHg; stage 3: SBP ≥ 180 mmHg or SBP ≥ 120 mmHg; BP: blood pressure

At baseline, the mean weight of participants was 76.4 ± 13.7 kg, and the mean body mass index (BMI) was 27.8 ± 4.9 kg/m^2^. Baseline mean office sitting systolic blood pressure (SBP) was 171.3 ± 14.3 mmHg, and the mean diastolic blood pressure (DBP) was 92.4 ± 9.6 mmHg. The median office pulse rate was 79 bpm [72-86 bpm]. Hypertension stages at baseline were classified as stage 1 (13.1%), stage 2 (54.0%), and stage 3 (32.9%). Blood pressure measurements were predominantly taken in the office (88.7%) (Table 1).

**Figure 2:**
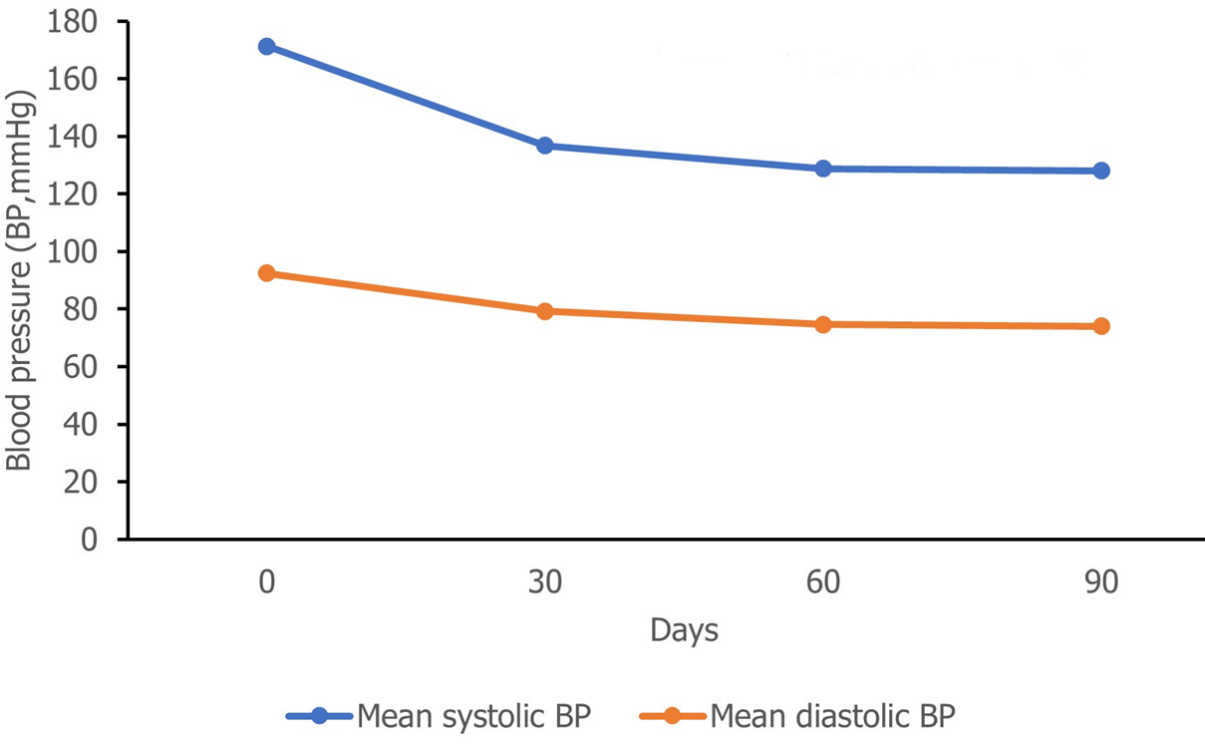
change in mean systolic and diastolic blood pressure across study visits

**Outcome data:** significant reductions in blood pressure were observed across all visits. At baseline, mean SBP and DBP were 171.3 mmHg (95% CI: 170.5-171.9) and 92.4 mmHg (95% CI: 91.9-92.9), respectively. On Day 30, significant reductions were observed in both SBP (-34.5 mmHg; 95% CI: -35.3 to -33.8, p<0.0001) and DBP (-13.1 mmHg; 95% CI: -13.6 to -12.6, p<0.0001). Further reductions were seen between day 30 and day 90, with additional decreases in SBP (-8.0 mmHg; 95% CI: -8.6 to -7.5, p<0.0001) and DBP (-4.6 mmHg; 95% CI: -5.0 to -4.2, p<0.0001) (Figure 2). The mean pulse rate also decreased from 79.4 bpm (95% CI: 78.9-79.9) to 72.2 bpm (95% CI: 71.9-72.6), with a mean difference of -7.2 bpm (95% CI: -7.7 to -6.7, p<0.0001).

**Figure 3:**
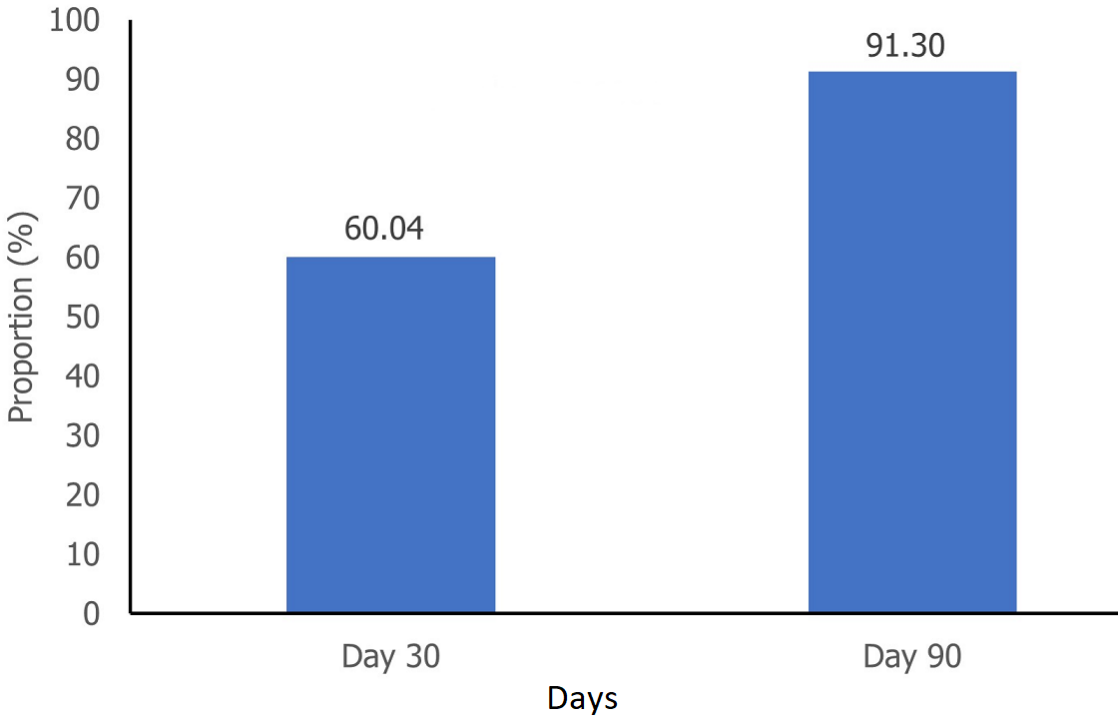
percentage of patients with blood pressure <140/90 mmHg on day 30 and day 90

**Proportion of participants achieving blood pressure control:** the proportion of participants achieving blood pressure control (<140/90 mmHg) increased significantly from 60.0% at day 30 to 91.3% at day 90 (p<0.0001) (Figure 3). More stringent targets, such as BP <130/80 mmHg, also showed significant improvements from 14.3% to 35.3%, respectively (both p<0.001) (see blood pressure changes between day 30 and day 60 in Annex 1).

**Main results:** after adjusting for covariates such as age, sex, duration of hypertension, smoking status, reported physical activity, diet, BMI, and comorbidities, the reductions in blood pressure remained significant. The adjusted mean difference in SBP was -29.3 mmHg (95% CI: -36.4 to -22.3, p<0.001), and the adjusted mean difference in DBP was -17.4 mmHg (95% CI: -22.2 to -12.5, p<0.001) (Table 2). The reductions in blood pressure from baseline were consistent across different stages of hypertension: 1) stage 1: mean SBP decreased by -26.1 mmHg (95% CI: -27.2 to -25.0, p<0.0001) and the mean DBP by -12.5 mmHg (95% CI: -13.6 to -11.3, p<0.0001); 2) stage 2: SBP decreased by -37.5 mmHg (95% CI: -38.2 to -36.9, p<0.0001) and DBP by -16.2 mmHg (95% CI: -16.8 to -15.5, p<0.0001); 3) stage 3: patients showed the most significant reduction with SBP by -57.3 mmHg (95% CI: -58.6 to -56, p<0.0001) and DBP by -22.6 mmHg (95% CI: -23.5 to -21.6, p<0.0001) (Annex 1).

**Table 2:** changes in systolic and diastolic blood pressure between baseline and day 90 among patients with hypertension uncontrolled on amlodipine monotherapy who received an amlodipine perindopril arginine-based strategy, Morocco, October 2023 to March 2024 (N=1,614)

Blood pressure (BP, mmHg)	Model 1	p	Model 2	p	Model 3	p
Systolic BP	-42.5(-43.3-41.8)	<0.001	-39.4(-44.2, -34.6)	<0.001	-29.3(-36.4, -22.3)	<0.001
Diastolic BP	-17.8(-18.3-17.2)	<0.001	-21.3(-24.6, -18.1)	<0.001	-17.4(-22.2, -12.5)	<0.001

Means and 95% confidence intervals are estimated using regression coefficients obtained from linear mixed-effects models; model 1 was unadjusted; model 2: adjusted for age, sex, hypertension years, smoking, reported physical activity, and healthy diet; model 3: additionally adjusted for BMI and other comorbidities (type 1 diabetes, type 2 diabetes, kidney disease, coronary heart disease, stroke, peripheral artery disease, and dyslipidaemia)

### Other analyses

**Treatment adherence and modification:** treatment adherence improved significantly over the study period. By day 90, the proportion of participants self-reporting forgetting to take their drugs decreased from 9.0% to 1.8% (p<0.0001). Instances of missed medication based on feeling better or worse also decreased significantly (all p<0.0001). Treatment adjustments were made primarily through dose adjustments and switching to the single-pill combination. By day 90, 97.7% of participants were on SPC (83.4% of study participants had their treatment modified to SPC based on their physician´s judgement on Day 30, and 36.4% of participants who were not on SPC on day 30 were put on SPC on day 60).

**Adverse events:** adverse events were reported by a small proportion of participants, with the overall number of reported adverse events decreasing over time. By day 90, only 0.25% of participants reported adverse events, including hypotension, fatigue, and constipation, among others (Table 3).

**Table 3:** adverse effects reported by participants during the study up to day 90 among patients with hypertension uncontrolled on amlodipine monotherapy treated with an amlodipine perindopril arginine-based strategy, Morocco, October 2023 to March 2024 (N=1,614)

Adverse events	Day 30 (n=1605 (%))	Day 60 (n=623 (%))	Day 90 (n=1598 (%))	p-value
Overall numbers	36 (2.24)	4 (0.64)	4 (0.25)	< 0.0001
Hypotension	3 (0.19)	0	1 (0.06)	
Palpitations	6 (0.37)	0	0	
Tachycardia or bradycardia	2 (0.12)	0	0	
Fatigue	8 (0.50)	0	1 (0.06)	
Vomiting	2 (0.12)	0	0	
Nausea	0	1 (0.16)	0	
Abdominal pain	3 (0.19)	0	0	
Constipation	1 (0.06)	1 (0.16)	1 (0.06)	
Dyspnoea	1 (0.06)	0	0	
Vertigo	4 (0.25)	0	0	
Somnolence	1 (0.06)	0	0	
Headaches	9 (0.56)	2 (0.32)	1 (0.06)	
Cough	8 (0.50)	0	0	
Pedal oedema	1 (0.06)	1 (0.16)	0	
Libido drop	1 (0.06)	0	0	

## Discussion

This observational study addresses a gap in real-world evidence by examining the effectiveness and safety of an amlodipine and perindopril arginine-based strategy among patients with hypertension in Morocco who were previously uncontrolled on amlodipine monotherapy. While international guidelines recommend ACE inhibitor/calcium channel blocker combinations, there is a paucity of real-world effectiveness data from North African healthcare settings. This study found significant reductions in both systolic and diastolic blood pressure over the 90-day period, consistent across different stages of hypertension. Furthermore, there was an increase in the proportion of patients who achieved blood pressure control, and self-reported treatment adherence improved. There was also a low incidence of adverse events on the combination therapy. These findings suggest the potential of an amlodipine and perindopril arginine-based strategy to effectively manage hypertension and improve patient outcomes in a real-world setting, particularly for the North African population.

Achieving reductions in blood pressure is crucial due to its significant impact on various health outcomes, and even small reductions can lead to substantial benefits. For instance, a meta-analysis highlighted that every 10-mmHg reduction in systolic blood pressure is associated with a significant decrease in cardiovascular disease and all-cause mortality [17]. Our study observed a 34.5-mmHg reduction in office SBP between day 0 and 30 and an additional 8-mmHg reduction between day 30 and 90, indicating the potential for the combination of amlodipine and perindopril arginine to contribute to cardiovascular outcomes in patients with hypertension in Morocco.

The combination of amlodipine and perindopril has consistently demonstrated antihypertensive efficacy in clinical trials such as the Anglo-Scandinavian Cardiac Outcomes Trial (ASCOT), significantly reducing cardiovascular morbidity and mortality in high-risk patients with hypertension up to 20 years of follow-up [18-20]. Moreover, the fixed-dose combination of perindopril and amlodipine has proven effective in reducing blood pressure and is well-tolerated in routine care [21,22]. This study aligns with these findings and extends their applicability to a North African context.

This study's results showed significant blood pressure reductions across all stages of hypertension. This finding is consistent with previous research demonstrating the effectiveness of combination therapy in lowering blood pressure, irrespective of the initial severity of hypertension [8]. This broad association suggests the potential utility of this treatment strategy for a wide range of patients with hypertension in Morocco. The substantial increase in the proportion of patients achieving blood pressure control (<140/90 mmHg) from 60.0% on day 30 to 91.3% on day 90 is noteworthy, as it is recommended that patients attain blood pressure control as soon as possible [4,23]. Modifying treatment to an SPC for blood pressure control is well supported by various studies. The amlodipine-perindopril in real settings (AMPERES) study shows that amlodipine and perindopril SPC therapy increases the rate of adherence and reduces the number of concomitant anti-hypertensive drugs in subjects previously treated with the same drugs as a two-pill combination [6,11,24,25].

The use of SPCs has been associated with cost savings through better blood pressure control, and increased adherence, requiring fewer physician visits and hospitalisations for cardiovascular events [13,26]. This is evidenced by a meta-analysis showing a 26% reduction in non-compliance rates compared to regimens based on free drug components [27]. This improved adherence is crucial for achieving optimal blood pressure control and preventing complications associated with uncontrolled hypertension. Adverse effects of antihypertensive medications can impact patient compliance and quality of life [28,29]. Therefore, selecting well-tolerated agents that promote good compliance is crucial for the cost-effective management of hypertension [30]. The low incidence of adverse events reported in this study (judged as not related to the medication as per protocol) is consistent with the established safety profile of amlodipine and perindopril arginine combination therapy. Previous studies have similarly reported minor side effects with this combination, confirming its tolerability and suitability for long-term hypertension management [31]. The low severity of the side effects observed further supports the combination's use in clinical practice.

Hypertension management is a multifaceted process involving various contributing factors such as age, sex, duration of hypertension, smoking status, physical activity, diet, BMI, and comorbidities [32-35]. In Morocco, where early diagnosis remains a challenge, these determinants may play a critical role in the effective management of hypertension [2]. This study also analysed the role of these factors on the observed outcomes with amlodipine and perindopril arginine combination therapy, showing that the combination was associated with blood pressure reductions even when adjusted for various risk factors. However, studies longer than 90 days would be needed in this population to assess cardiovascular outcomes.

**Strengths and limitations of the study:** this observational study's large sample size and multicentre design increase the generalisability of the findings within the Moroccan context, providing data from a real-world setting that offers practical insights into the effectiveness and adherence of the treatment regimen. The follow-up and data collection on blood pressure, adherence, and adverse events further strengthen the study.

However, several important limitations must be acknowledged. As an observational study, patient enrollment was based on physician discretion, which may have introduced selection bias for patients more likely to benefit from combination therapy and limited causal inference. The absence of a control group means we cannot definitively attribute the observed BP reductions solely to the medication combination. Adherence was assessed solely through self-report without objective measures such as pill counts or prescription refill data, which may overestimate actual medication-taking behaviour. Limited use of ambulatory blood pressure monitoring could restrict detailed insights into blood pressure variations. Additionally, although standardized BP measurement procedures were recommended, variation in equipment and routine clinical practice across centres may have introduced measurement error.

These limitations may have influenced the magnitude of the observed effects. Physician-driven patient selection and reliance on self-reported adherence are likely to bias the results toward overestimating effectiveness, while variability in BP measurement techniques across centres would introduce non-differential measurement error, biasing results toward the null. Overall, the direction of these biases is mixed but unlikely to fully account for the substantial BP reductions observed.

**Recommendations for future research:** future research should focus on evaluating the long-term cardiovascular outcomes and survival rates associated with an amlodipine and perindopril arginine-based combination therapy. Conducting randomised controlled trials will help establish causality and compare this therapy with other antihypertensive treatment strategies. Studies should explore the treatment's effectiveness across diverse populations and investigate the underlying pharmacodynamic and pharmacokinetic mechanisms. These will further validate these findings and inform clinical practice.

## Conclusion

This study suggests an association between an amlodipine and perindopril arginine-based strategy and improved blood pressure control among patients with hypertension in Morocco who were uncontrolled on amlodipine monotherapy. We observed a marked increase in the proportion of patients treated with the combination therapy of amlodipine and perindopril arginine. Self-reported treatment adherence improved, and a favourable safety profile was also observed with SPC, highlighting the therapy's potential for enhancing hypertension management. These findings support the use of an amlodipine and perindopril arginine-based strategy as a viable and effective option for achieving optimal blood pressure control in patients with hypertension who are uncontrolled on amlodipine monotherapy.

### What is known about this topic

- International guidelines recommend calcium channel blockers as first-line agents in African populations, with combination therapy advised when monotherapy fails;
- The amlodipine-perindopril single-pill combination has shown superior efficacy and safety in clinical trials.

### What this study adds

- We found that adding perindopril to amlodipine in patients uncontrolled on monotherapy led to significant reductions in SBP and DBP over 90 days;
- We demonstrated that a high proportion of patients achieved BP control, and that transitioning to single-pill combinations was feasible and associated with good tolerability in real-world Moroccan clinical practice.

## Annex

**Annex 1**: supplementary materials (PDF- 192KB)
